# Decoction regulating phytochemicals’ micromorphology changes and anti-inflammation activity enhancements originated from herb medicine supermolecules

**DOI:** 10.1186/s13020-023-00864-z

**Published:** 2024-01-26

**Authors:** Luping Yang, Xiang Zhang, Zhijia Wang, Xiaoyu Lin, Yaozhi Zhang, Jihui Lu, Linying Wu, Shuchang Yao, Wenguang Jing, Xuemei Huang, Penglong Wang

**Affiliations:** 1 School of Chinese Pharmacy, Beijing University of Chinese Medicine, Beijing, 102488 China; 2 National Institutes for Food and Drug Control, Beijing, 100050 China

**Keywords:** Mahuang Fuzi decoction, Supermolecules, Decoction, Inflammation, Metabolomics

## Abstract

**Background:**

Mahuang Fuzi decoction (MGF) is composed of three herb medicines that has been clinically used to treat inflammatory diseases for a long history. At present, more and more active phytochemicals’ aggregations have been found during the thermodynamic process of herb medicine decoction, and revealing the clinical efficacy of herb medicine through supramolecular strategies is the focus of current research. However, it is not clear whether decoction induced supermolecules’ morphological changes to modify activity.

**Methods:**

Dynamic light scattering (DLS) and field emission scanning electron microscopy (FESEM) were used to analyze the micromorphology of MGF, MGF SA (MGF supermolecules), and MIX (physical mixture of MGF single decoction). The interaction and thermodynamic parameters of single herbs in a decoction were investigated by Isothermal titration calorimetry (ITC). The phytochemicals were systematically analyzed by ultra high performance liquid chromatography-Q Exactive hybrid quadrupole-orbitrap high-resolution accurate mass spectrometry (UHPLC-Q-Orbitrap HRMS). Under the safe dose on RAW264.7 cells, NO, IL-6 and TNF-α were determined by Enzyme-Linked ImmunoSorbent Assay (ELISA) method. NF-κB p65 translocation from the cytoplasm into the nucleus was examined using the immunofluorescence assay and the western blot, respectively. Furthermore, Metabolomics was used to discover potential biomarkers and the associated metabolic pathways of MGF SA treatment.

**Results:**

There were nanoscale aggregations in MGF, and the micromorphology of the extracted MGF SA consisted of uniform particles; while the MIX micromorphology had no uniformity. ITC showed that the interaction MH-GC and FZ-GC were a spontaneous exothermic reaction, indicating that their phytochemicals had the property of self-assembly. Though the micromorphology between MGF, MGF SA, and MIX was obviously different, UHPLC-Q-Orbitrap HRMS results displayed that the main phytochemicals of MGF and MIX had nearly the same components. Interestingly, MGF and MGF SA could significantly inhibit the production of NO, and had better inhibition effect on the expression of nuclear protein NF-κB p65 than MIX, among which MGF SA had the best effect. Further investigation indicated that the perturbance of metabolic profiling in RAW264.7 inflammatory cells was obviously reversed by MGF SA.

**Conclusions:**

The decoction enriched the key active phytochemicals and regulated the formation of homogeneous nanoparticles in MGF SA. The supermolecules in MGF SA significantly enhanced its anti-inflammatory activity, primarily affecting the NF-κB signaling pathway and the biosynthesis and metabolism of arginine in RAW264.7 inflammatory cells. Current study displayed that co-decocting herbal medicine were beneficial to the treatment of diseases than the mixture of the single herbs’ extraction.

**Graphical Abstract:**

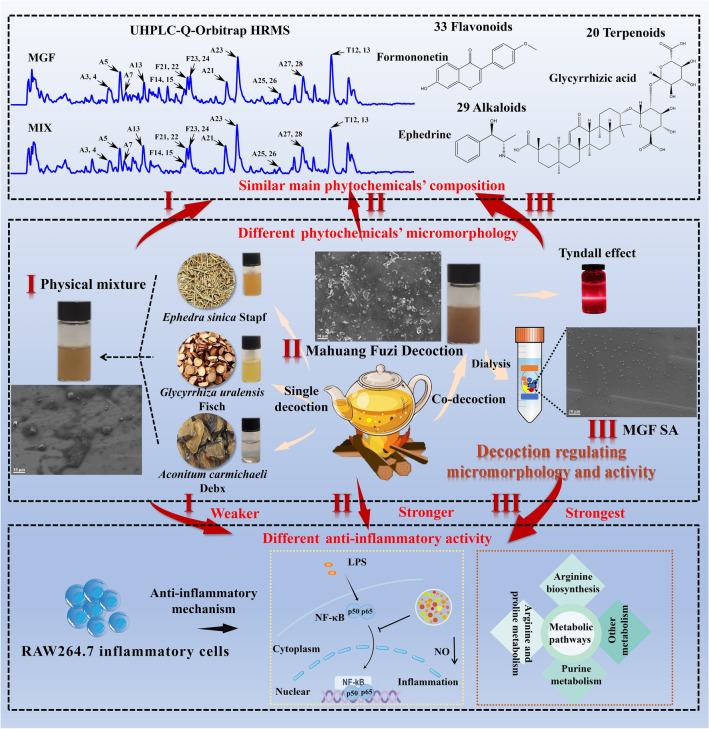

## Introduction

Herbal medicine decoction has been a crucial method for preventing and treating illnesses for millennia, and remains one of the most prevalent forms of medication used in clinical settings [1–5]. Several herb medicines combined together can not only enhance clinical efficacy, but also reduce adverse reactions [6, 7]. However, the material basis of the effect of herb medicine decoction had always been the hotspots and difficulties in academic research. At present, researchers have found that there are various of bioactive aggregates in herb medicine decoction. Especially with the wide application of supramolecular chemistry, it is found that the in-depth study of aggregates shows a broad prospect in elucidating the effective mechanism of herb medicine decoction and developing nano-drugs [8, 9]. Supramolecular chemistry was proposed by Nobel Prize winner Jean-Marie Len in 1973. It typically refers to the combine of two or more molecules through intermolecular interactions, resulting in intricate and well-organized aggregates [10, 11]. At the same time, with the popularization of microscopic morphology research techniques, the study of supramolecular aggregates in herb medicine decoction has made some breakthroughs. For example, our laboratory has identified supermolecules present in the decoction of *Rhei Radix et Rhizoma* and *Coptidis Rhizoma*, namely emodin and coptisine, have the ability to form nanoparticles (NPs) with a size of approximately 50 nm, nanofibers can be formed by rhein and coptidine [12]; the supramolecular components of *Glycyrrhiza uralensis* Fisch (GC) and *Coptidis Rhizoma* co-decoction were important substances to exert their antibacterial effects [13]; NPs with different sizes in the Huanglian Jiedu decoction showed good inhibitory effect on bacteria [14]. In addition, Zhou et al. [15] was able to effectively isolate colloidal nanoparticles from Maxing Shigan decoction and found that these particles were linked to ephedrine and pseudoephedrine. The supramolecules present in the herb medicine decoction serve as a significant material foundation for the therapeutic effects of the substance.

At present, due to the problems of inconveniently carrying decoction and the low utilization rate of herb medicines, modern medicine has changed traditional decoction into granules, extracts, and other new dosage forms [16]. Nevertheless, compared to the thousands of years of conventional decoction, they had been the subject of significant and contentious debate because of novel dosage forms that lack the method of co-decocting combination herb medicines. By comparing the difference between formula granules and combined decoction of *Coptidis Rhizome* and *Evodiae Fructus*, Quan [17] found that the formula granules could not reproduce the trend of influence of combined decoction of herb medicine on proportion and component content. Huang [18] found that the thermodynamic heating process promoted the assembly of berberine and baicalin to form supermolecule’ nanospheres, which produced a better antibacterial effect than the physical mixture. These results indicate that herb medicine co-decoction is one of the necessary conditions for the formation of herb medicine supermolecules, which affects the biological activity of clinical herb medicine. However, there are few reports on the effect of co-decocting on the supermolecules’ micromorphology and biological activity of herb medicine, and the effect of supermolecules’ micromorphology changes on its biological activity under decoction regulation is not clear.

Mahuang Fuzi decoction (MGF), as a classic prescription in the Synopsis of Prescriptions of the Golden Chamber used clinically for 1800 years, consists of three kinds of Chinese herbs, namely *Ephedra sinica* Stapf (MH), GC, *Aconitum carmichaeli* Debx (FZ). Among them, MH as a traditional medicine used in Oriental medicine for thousands of years, can effectively reduce inflammation, lower fever and relieve pain [19–22]. FZ is first recorded in Shennong Classic of Materia Medica and its main function is analgesic, anti-inflammatory, antibiotic, antipyretic, cardiotonic, etc. [23, 24]. GC is a very famous ancient herbal medicine, often used in combination with other herb medicines [25–27]. This prescription has been reported for inflammatory treatment, although its anti-inflammatory effects’ molecular mechanisms are still poorly understood [28].

**Fig. 1:**
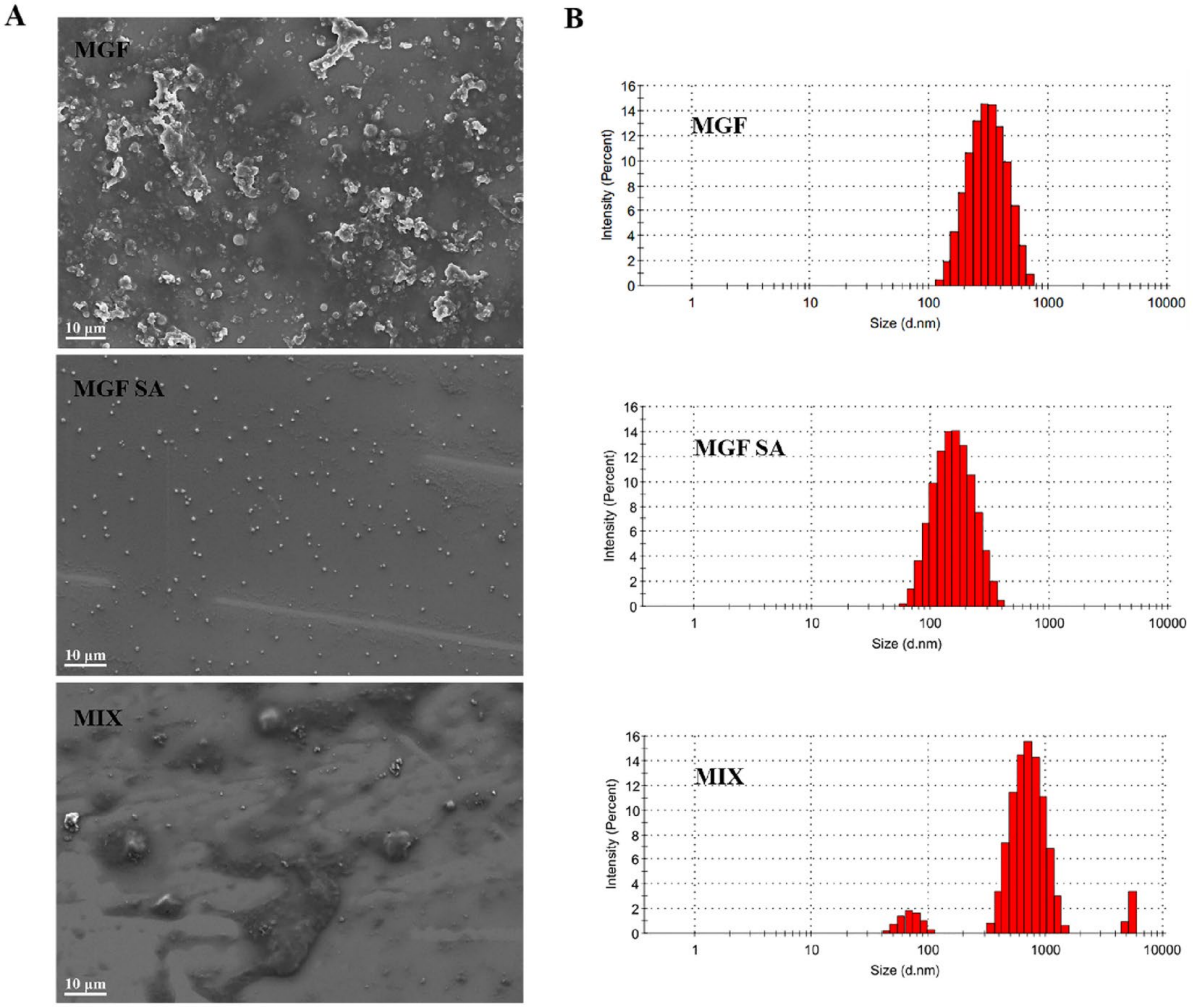
Characterization of MGF, MGF SA and MIX. **A** FESEM image of MGF, MGF SA and MIX. **B** Size distribution of MGF, MGF SA and MIX

In this study, based on decoction and supramolecular strategies, we observed the micromorphological characteristics of MGF co-decoction, the supermolecules from co-decoction (MGF SA) and the physical mixture of three herbs single decoction (MIX), respectively. The interaction and thermodynamic parameters of MH, FZ and GC were investigated by Isothermal titration calorimetry (ITC). Then, using UHPLC-Q-Orbitrap HRMS technologies, the phytochemicals were analyzed. In addition, whether decoction induced supermolecules’ micromorphology changes to modify activity was further discussed in an inflammatory model on RAW264.7 cells. Furthermore, the nuclear transcription factor-κB (NF-κB) signaling pathway and metabolic profiling the anti-inflammatory mechanism of MGF, MGF SA and MIX were explored, respectively. As shown in Fig. 1, decoction regulating phytochemicals’ micromorphology and anti-inflammation activity changes originated from herb medicine supermolecules can offer a fresh viewpoint for the fundamental study of the pharmacodynamics of MGF, which can prompt the herb medicine decoction’s clinical efficacy.

## Materials and methods

### Materials

Ephedra *sinica Stapf* (MH), Glycyrrhiza *uralensis Fisch* (GC), and Aconitum carmichaeli Debx (FZ) were obtained from Tongrentang in Beijing, China (batch number: 220260591, 220360851, 22080204). 3-(4,5-dimethylthiaxolone-2-yl)-2,5-diphenyltetrazoliumbromide (MTT) and Nitric Oxide (NO) Assay Kit were supplied by Bairuiji Biotechnology Co. (Beijing, China). Nuclear and cytoplasmic extraction kit and Hoechst 33342 were purchased by Beyotime Biotechnology Co. (Shanghai, China). Anti-NF-κB p65 was obtained from Abcam (Cambridge, UK). Secondary AlexaFluor488 antibody was supplied from Servicebio (Wuhan, China).

### Sample preparation

MGF was obtained by soaking 9 g MH, 6 g GC and 3 g FZ in 10 times deionized water for 30 min, and then boiling for 1 h. The MGF was subjected to hot centrifugation, followed by dialysis in deionized water for 12 h using a dialysis bag with an interception weight of 3500, resulting in the production of MGF SA. MH, GC and FZ were boiled separately and mixed to obtain MIX.

### Cell culture

The RAW264.7 cells used in this study were provided by Cell Bank, Chinese Academy of Sciences, Shanghai. Mouse macrophage cell line RAW264.7 was cultured in DMEM complete medium containing antibiotics (100 U/mL penicillin and 100 U/mL streptomycin) and 10% heat-inactivated FBS at 37 °C, 5% CO_2_ conditions in a humidified incubator. The cells were scraped off by Cell scraper, centrifuged at 1000 rpm for 3 min, resuspended with the complete culture medium, and then transferred to a new culture flask.

### Field emission scanning electron microscopy (FESEM) observation

After spraying gold on the silicon wafer, the 2.5 μL MGF, MGF SA, and MIX solution were dropped onto the surface of the silicon wafer respectively. Then, FESEM (ZEISS, Oberkochen, Germany) was used to examine the micromorphology of MGF, MGF SA, and MIX.

### Dynamic light scattering (DLS) method observation

Each sample was diluted with deionized water and transferred to a colorimetric dish. The particle size of MGF, MGF SA and MIX were examined by Malvern particle size analyzer (Malvern Panaco, Maleven, UK).

### Isothermal titration calorimetry (ITC) analysis

MF, GC and FZ decoction were filtered by 0.45 μm microporous membrane, and then characterize the interaction among single decoction of MH, GC and FZ, in which deionized water were used as the control groups by NANO ITC (TA Instruments, Delaware, USA). The following instrument settings were made: 250 rpm stirring, 25 °C titration temperature, and 180 s titration interval.

### Analysis was performed by UHPLC-Q-Orbitrap HRMS

UHPLC-Q-Orbitrap HRMS analysis was performed using a UltiMate 3000 liquid chromatographic system and coupled with a Q Exactive quadrupole-Orbitrap high-resolution mass spectrometry (Thermo Fisher Scientific, Massachusetts, USA). The analysis was carried out on a TC-C18 column (4.6 mm × 250 mm, 5 μm, Agilent). The mobile phase was made up of 0.1% (v/v) aqueous formic acid solution (A) and acetonitrile (B). 0–30 min, 4–98% B were the gradient elution conditions. The electrospray ionization (ESI) ion source was used to gather data. The electrospray ionization (ESI) ion source was used to gather data. The scan mode in Full MS/dd-MS^2^ mode. The mass range was scanned from 150 to 1500 m*/z*. Data was analyzed by Xcalibur version 4.1 software.

### MTT assay

The RAW264.7 cells (1 × 10^5^ cells/mL) were cultured 24 h in an incubator with CO_2_ concentration of 5% at 37 °C. Weighing an appropriate amount of freeze-dried powder of the decoction, we dissolve it in boiling distilled water and vortex it, so that it is fully dissolved. Then, the medium containing different concentrations of MGF, MGF SA, and MIX were changed so that the final concentration of MGF, MGF SA, and MIX powder were 2000, 1000, 500, 250, 125, and 62.5 mg/L. Each concentration had six replicate wells, while set up a blank control group. After incubation with 5 g/L of MTT per well, 200 µL of DMSO was added, and absorbance was finally measured at 490 nm.

### Measurements of pro-inflammatory cytokines in supernatant

Enzyme-Linked ImmunoSorbent Assay (ELISA) was used to evaluate the NO, IL-6 and TNF-α levels in the culture supernatant of RAW264.7 cells. The cells were divided into groups of normal, model, MGF, MGF SA and MIX. For the MGF, MGF SA, and MIX groups, 200 µL of medium with concentrations of 500 mg/L of MGF, MGF SA, and MIX, respectively, and 1 µg/mL lipopolysaccharide (LPS) were added to a 96-well plate and cultivated for 24 h. The model group was added with medium containing LPS, while the normal group was only given medium. According to the ELISA kit instructions, the optical density (OD) was measured by the enzyme-labeled instrument.

### NF-κB p65 nuclear translocation immunofluorescence assay

The cells were fixed with freshly prepared 4% paraformaldehyde for 20 min and then permeabilized in 0.5% Triton X-100 for 20 min. The permeabilized cells were subjected to three washes using PBS. Following incubation with the NF-κB p65 antibody and a secondary AlexaFluor488 antibody, the nuclei were stained with Hoechst 33342 at 37 °C for 10 min in a dark environment. A luminescence microscope was utilized to capture images (Nikon, Tokyo, Japan).

### Expression of total, nuclear and cytoplasmic proteins of NF-κB p65

The cells of each group were washed with PBS for 3 times, then corresponding proteins were extracted according to the instructions of the nuclear and cytoplasmic extraction kit. The protein concentrations of the cells were measured using bicinchoninic acid (BCA) method. The separated proteins by sodium dodecyl sulfate–polyacrylamide gel electrophoresis (SDS-PAGE) were transferred onto PVDF membranes. The membranes were incubated with primary antibodies against NF-κB p65 and β-actin overnight at 4 °C, followed by incubation with HRP-conjugated goat anti-rabbit IgG (H+L) secondary antibodies. Finally, protein bands were visualized using an ECL autoradiography kit.

### Untargeted metabolomics analysis

In this study, organic solvent extraction method was used to extract metabolites from cells in each group, which achieved the purpose of protein removal and maximum metabolite extraction. After nitrogen drying, each sample was redissolved with 200 μL of methanol for metabolomics analysis of intracellular substances. An UltiMate 3000 high performance LC system coupled to Q Exactive MS was utilized for metabolic profiling [29]. Finally, statistical modeling and analysis of LC–MS data were constructed using SIMCA-14.1 software and MetaboAnalyst analysis platform.

### Statistical analysis

The experimental data were expressed as mean ± standard deviation. Statistical comparison among multiple groups was carried out by one-way analysis of variance (one-way ANOVA) followed by LSD test (data with homogeneity of variance) in IBM SPSS 26.0. The variances were considered to have statistical significance at *p* < 0.05 or *p* < 0.01, respectively.

## Results and discussion

### Micromorphology and characterization of MGF, MGF SA and MIX

**Scheme 1:**
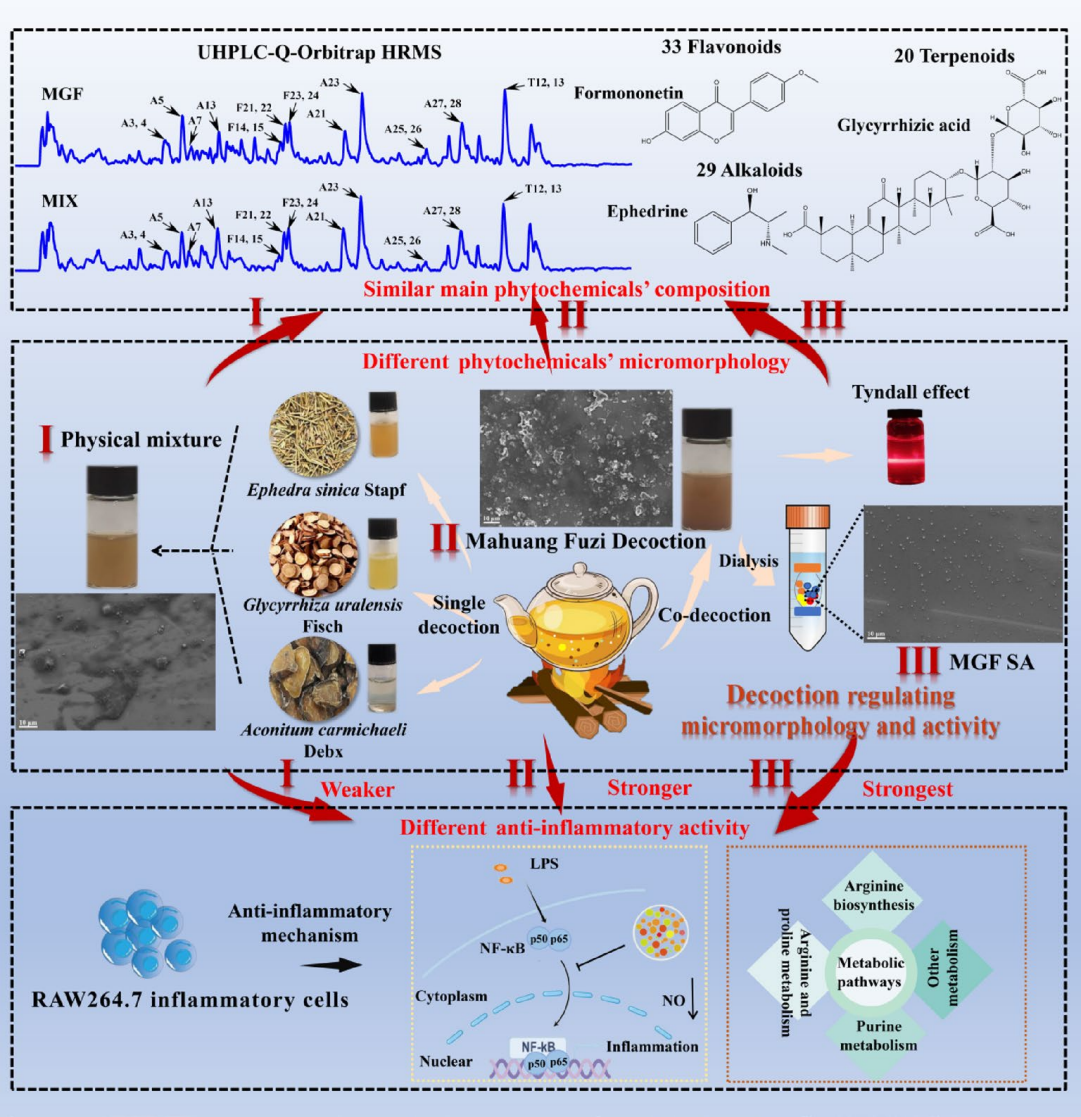
Decoction regulating phytochemicals’ micromorphology and anti-inflammation activity changes originated from herb medicine supermolecules

As shown in Scheme 1, the turbidity of MGF was evident, with aggregations being observed, but there was no apparent settling. In contrast, after being decocted separately, the single decoction of MH, FZ, or GC exhibited relatively clear appearance. This observation indicated that the phytochemicals present in the co-decoction did not exist as individual compounds, but rather interact with one another, resulting in a complex and heterogeneous appearance of the decoction. In the meantime, we found that the MGF had obvious Tyndall phenomenon (Scheme 1), which indicated that there were nano-form aggregations in the decoction. The FESEM results of MGF and MIX further indicated that the active phytochemicals would spontaneously assemble into supermolecules with certain regular micromorphology during decocting (Fig. 1A). These results indicated that decoction could regulate the supramolecules’ micromorphology of herb medicine decoction. Subsequently, the MGF SA was extracted by dialysis. It was worth noting that MGF SA was almost uniform spherical particles without agglomeration (Fig. 1A). Obviously, the micromorphology of the MGF SA was more uniform than that of the co-decoction. In addition, the size distribution experiments of MGF, MGF SA and MIX alsoverified this trend. As shown in Fig. 1B, the average diameter of these particles were about 285.8 nm, 123.5 nm and 666.0 nm, respectively, which were consistent with FESEM results.

### Isothermal titration calorimetry analysis between the herb medicine

**Fig. 2:**
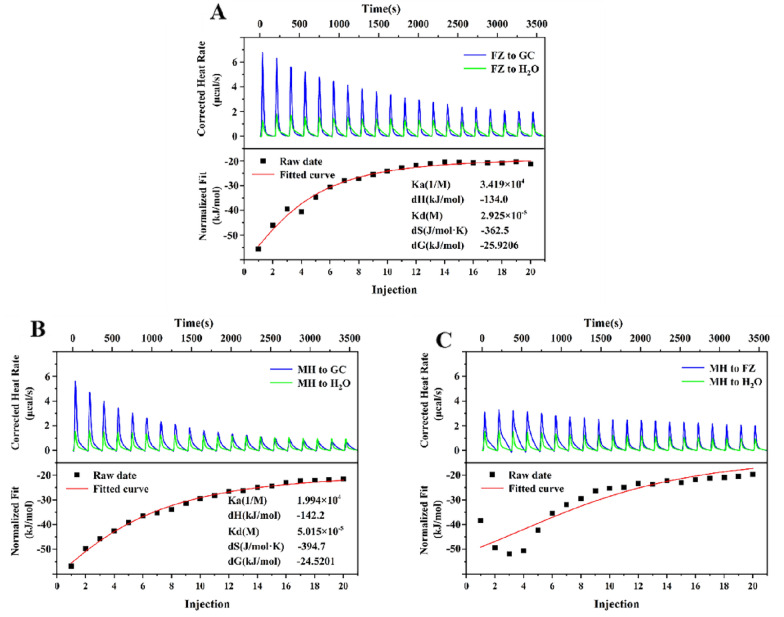
ITC characterization. **A** FZ-titrated GC. **B** MH-titrated GC. **C** MH-titrated FZ

ITC technology was used to continue investigate the thermodynamic changes between herb medicine to determine whether there was interaction between herb medicine. Figure 2 showed the results of mutual titration curves and fitting curves of herb medicine in MGF. The titration energy peaks of FZ-GC and MH-GC faced upward, and the fitting curves were approximately S-shaped, indicating that there was interaction between FZ, MH and GC during titration, and the reaction was exothermic. Among them, the energy decline trend of FZ-GC was the most obvious, followed by MH-GC, and MH-FZ showed a less obvious energy decline trend. By using Nano Analyze software, the thermodynamic parameters were obtained after fitting the data. It was found that the thermodynamic parameters of FZ-GC and MH-GC were both dH < 0, − TdS > 0, |dH| >|− TdS|, dG < 0, indicating that the active components of MH, FZ had non-covalent interaction with the active components of GC, such as hydrogen bond and electrostatic attraction, rather than aggregation caused by physical adsorption.

### Identification of the phytochemicals in the MGF, MGF SA and MIX

**Fig. 3:**
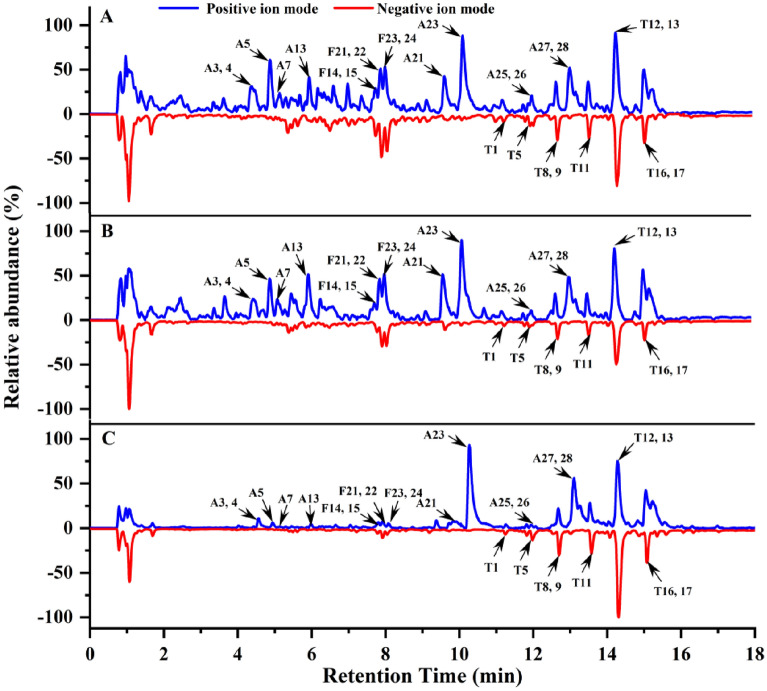
Total ion flow diagram. **A** MGF. **B** MIX. **C** MGF SA

To further determine the phytochemicals of MGF, MGF SA and MIX, UHPLC-Q-Orbitrap HRMS analysis was conducted. Figure 3 was the total ion flow diagram of MGF, MGF SA and MIX, respectively. In this study, totally 82 phytochemicals had been identified, including 29 alkaloids, 33 flavonoids, 20 terpenoids (Tables 1, 2 and 3, respectively). And it could be seen that the main phytochemicals’ composition of MGF, MGF SA and MIX was similar. Especially, the chemical content of MGF and MIX was almost the same, but the micromorphology was quite different, suggesting that the difference in micromorphology was not caused by the production of new phytochemicals in the decocting process, but decoction induced the molecular rearrangement and assembly of phytochemicals. As could be seen from Fig. 3 that MGF SA had enriched the key active phytochemicals in supramolecules induced by decoction.

**Table 1:** The Alkaloids identified in the MGF, MGF SA and MIX

NO	t_R_ (min)	Compound	Formula	Precursor ion				Fragment ion (*m/z*)	Source MGF(Q)/MGF SA(T)/MIX(X)
				Identity	Theoretical (*m/z*)	Experimental (*m/z*)	Mass accuracy (Δppm)		
A1	1.40	Transtorine	C_10_H_7_NO_3_	[M+H]^+^	190.0498	190.0498	0	162.1122	Q/T/X
A2	3.74	Delavaconine	C_22_H_35_NO_5_	[M+H]^+^	394.2587	394.2578	− 2.43	376.2466, 340.1384	Q/T/X
A3/A4	4.41	Ephedrine/pseudoephedrine	C_10_H_15_NO	[M+H]^+^	166.1226	166.1224	− 1.14	148.1116, 133.0884, 117.0699, 91.0545	Q/T/X
A5	4.81	Mesaconine	C_24_H_39_NO_9_	[M+H]^+^	486.2697	486.2683	− 2.91	468.2593, 454.2429, 436.2316, 422.2151	Q/T/X
A6	5.08	Isotalatizidine	C_23_H_37_NO_5_	[M+H]^+^	408.2744	408.2733	− 2.69	390.2627	Q/T/X
A7	5.17	Karakoline	C_22_H_35_NO_4_	[M+H]^+^	378.2638	378.2628	− 2.65	360.2522	Q/T/X
A8	5.27	Aconine	C_25_H_41_NO_9_	[M+H]^+^	500.2854	500.2839	− 2.99	468.2596, 418.2240	Q/T/X
A9	5.30	Ephedradine B	C_29_H_38_N_4_O_5_	[M+H]^+^	523.2914	523.2913	− 0.22	493.2804	Q/T/X
A10	5.61	Songorine	C_22_H_31_NO_3_	[M+H]^+^	358.2376	358.2365	− 3.07	340.2261	Q/T/X
A11	5.74	Hetisine	C_20_H_27_NO_3_	[M+H]^+^	330.2063	330.2052	− 3.45	312.1954	Q/T/X
A12	5.83	Senbusine A	C_23_H_37_NO_6_	[M+H]^+^	424.2693	424.2691	− 0.55	374.2314, 342.2037	Q/T/X
A13	5.86	Hypaconine	C_24_H_39_NO_8_	[M+H]^+^	470.2748	470.2740	− 1.68	438.2475, 406.2222	Q/T/X
A14	6.08	Fuziline	C_24_H_39_NO_7_	[M+H]^+^	454.2799	454.2794	− 3.27	436.2679, 404.2419	Q/T/X
A15	6.36	Neoline	C_24_H_39_NO_6_	[M+H]^+^	438.2850	438.2848	− 3.39	420.2750, 388.2475, 356.2212	Q/T/X
A16	6.37	6-Methoxykynurenic acid	C_11_H_9_NO_4_	[M+H]^+^	220.0604	220.0603	− 0.38	190.1309	Q/T/X
A17	7.07	Talatizamine	C_24_H_39_NO_5_	[M+H]^+^	422.2900	422.2892	− 2.08	390.2628	Q/T/X
A18	7.68	Chasmanine	C_25_H_41_NO_6_	[M+H]^+^	452.3006	452.2995	− 2.50	420.2732, 388.2746	Q/T/X
A19	8.19	14-Acetyltalatizamine	C_26_H_41_NO_6_	[M+H]^+^	464.3006	464.3002	− 3.02	432.2731	Q/T/X
A20	8.32	14-Benzoy-10-OH-mesaconine	C_31_H_43_NO_11_	[M+H]^+^	606.2908	606.2891	− 2.91	556.2535	Q/T/X
A21	9.64	Benzoylmesaconine	C_31_H_43_NO_10_	[M+H]^+^	590.2959	590.2948	− 1.88	558.2239, 526.4912, 508.3276, 482.2066	Q/T/X
A22	10.23	Benzoylaconine	C_32_H_45_NO_10_	[M+H]^+^	604.3116	604.3099	− 2.78	572.4022, 540.1622, 522.1387	Q/T/X
A23	10.68	Benzoylhypaconine	C_31_H_43_NO_9_	[M+H]^+^	574.3010	574.2991	− 3.25	542.2719, 510.2502	Q/T/X
A24	10.97	Pyromesaconitine	C_31_H_41_NO_9_	[M+H]^+^	572.2854	572.2852	− 0.32	540.1609, 522.1375	Q/T/X
A25	12.00	Mesaconitine	C_33_H_45_NO_11_	[M+H]^+^	632.3065	632.3060	− 0.70	582.2117, 572.4024, 540.1611, 522.3276, 512.4753	Q/T/X
A26	12.03	Aconifine	C_34_H_47_NO_12_	[M+H]^+^	662.3171	662.3161	− 1.42	612.1836, 602.4131, 584.5106, 542.5142	Q/T/X
A27	12.92	Aconitine	C_34_H_47_NO_11_	[M+H]^+^	646.3221	646.3201	− 3.18	538.1632, 526.4910	Q/T/X
A28	13.08	Hypaconitine	C_33_H_45_NO_10_	[M+H]^+^	616.3116	616.3100	− 2.53	556.2861, 496.3461	Q/T/X
A29	13.95	Deoxyaconitine	C_34_H_47_NO_10_	[M+H]^+^	630.3272	630.3265	− 1.16	598.2984, 570.3065, 538.2811, 510.2838, 506.2526	Q/T/X

**Table 2:** The flavonoids identified in the MGF, MGF SA and MIX

NO	t_R_ (min)	Compound	Formula	Precursor ion				Fragment ion (*m/z*)	Source MGF(Q)/MGF SA(T)/MIX(X)
				Identity	Theoretical (*m/z*)	Experimental (*m/z*)	Mass accuracy (Δppm)		
F1/F2	0.80	Epicatechin/catechin	C_15_H_14_O_6_	[M−H]^−^	289.0706	289.0715	2.99	151.6712, 137.0234	Q/T/X
F3	3.61	Vestitol	C_16_H_16_O_4_	[M+H]^+^	273.1121	273.1117	− 1.63	243.8780, 123.0553	Q/T/X
F4	5.67	Herbacetin	C_15_H_10_O_7_	[M+H]^+^	303.0499	303.0493	− 0.57	195.0912	Q/T/X
F5	5.88	Rutin	C_27_H_30_O_16_	[M+H]^+^	611.1606	611.1601	− 1.93	303.1339	Q/T/X
F6	6.38	Vicenin II	C_27_H_30_O_15_	[M+H]^+^	595.1657	595.1650	− 1.18	271.0952	Q/T/X
F7	6.86	Echinatin	C_16_H_14_O_4_	[M+H]^+^	271.0964	271.0968	0.31	239.1277, 151.1116	Q/T/X
F8	7.04	Schaftoside	C_26_H_28_O_14_	[M−H]^−^	563.1395	563.1377	− 3.25	431.1900, 401.1455, 268.8465	Q/T/X
F9	7.08	Daidzein	C_15_H_10_O_4_	[M+H]^+^	255.0651	255.0649	− 1.11	137.0231	Q/T/X
F10	7.34	Kumatakenin	C_17_H_14_O_6_	[M+H]^+^	315.0863	315.0860	− 0.71	167.0127	Q/T/X
F11/F12	7.39	Quercetin 3-*O*-galactoside/herbacetin 7-glucoside	C_21_H_20_O_12_	[M+H]^+^	465.1027	465.1025	− 0.52	303.0495	Q/T/X
F13	7.66	Apigenin 7-*O*-glucoside	C_21_H_20_O_10_	[M+H]^+^	433.1129	433.1113	− 3.54	271.0590	Q/T/X
F14/F15	7.69	Liquiritigenin/Isoliquiritigenin	C_15_H_12_O_4_	[M+H]^+^	257.0808	257.0802	− 0.54	137.0229, 123.0435	Q/T/X
F16	7.72	Isovitexin2-*O*-rhamnoside	C_27_H_30_O_14_	[M−H]^−^	577.1551	577.1534	− 3.01	413.0857, 395.0730, 293.0441	Q/T/X
F17/F18	7.76	Violanthin/isoviolanthin	C_27_H_30_O_14_	[M+H]^+^	579.1708	579.1701	− 1.26	433.1123, 271.0789	Q/T/X
F19	7.81	Calycosin	C_16_H_12_O_5_	[M+H]^+^	285.0757	285.0755	− 0.66	269.0440	Q/T/X
F20	7.81	Licoflavone A	C_20_H_18_O_4_	[M+H]^+^	323.1277	323.1280	0.23	205.0856, 163.0383, 161.0023	Q/T/X
F21	7.93	Naringenin	C_15_H_12_O_5_	[M+H]^+^	273.0757	273.0756	− 0.13	123.0439	Q/T/X
F22	7.95	Kaempferol	C_15_H_10_O_6_	[M+H]^+^	287.0550	287.0549	− 0.05	137.0230	Q/T/X
F23	8.03	Licochalcoe B	C_16_H_14_O_5_	[M+H]^+^	287.0914	287.0902	− 1.19	193.0849, 167.1064	Q/T/X
F24	8.04	Isoliquiritin apioside	C_26_H_30_O_13_	[M−H]^−^	549.1602	549.1586	− 1.69	417.1192, 255.0661	Q/T/X
F25	8.99	Hesperidin	C_28_H_34_O_15_	[M−H]^−^	609.1813	609.1799	− 1.40	463.2561, 445.0788, 283.0615	Q/T/X
F26	9.68	Liquiritin apioside	C_26_H_30_O_13_	[M+H]^+^	551.1759	551.1747	− 1.17	419.1331	Q/T/X
F27	9.86	Swertisin	C_22_H_22_O_10_	[M+H]^+^	447.1285	447.1281	− 0.46	329.1204, 242.1745	Q/T/X
F28	9.94	Liquiritin	C_21_H_22_O_9_	[M+H]^+^	419.1336	419.1324	− 2.88	257.0798, 239.0695, 211.0746, 137.0229	Q/T/X
F29	9.98	Ononin	C_22_H_22_O_9_	[M+H]^+^	431.1336	431.1326	− 2.38	269.0801	Q/T/X
F30	9.98	Formononetin	C_16_H_12_O_4_	[M+H]^+^	269.0808	269.0800	− 2.80	254.0557, 226.1584, 213.0910, 137.0230	Q/T/X
F31	10.11	Apigenin	C_15_H_10_O_5_	[M−H]^−^	269.0444	269.0443	− 0.40	241.0254, 176.0345	Q/T/X
F32	10.90	Eurycarpin A	C_20_H_18_O_5_	[M+H]^+^	339.1227	339.1242	4.45	325.1068, 203.1429, 247.1325	Q/T/X
F33	13.27	Tricin	C_17_H_14_O_7_	[M−H]^−^	329.0655	329.0660	1.30	315.2176	Q/T/X

**Table 3:** The Terpenoids identified in the MGF, MGF SA and MIX

NO	t_R_ (min)	Compound	Formula	Precursor ion				Fragment ion (*m/z*)	Source MGF(Q)/MGF SA(T)/MIX(X)
				Identity	Theoretical (*m/z*)	Experimental (*m/z*)	Mass accuracy (Δppm)		
T1	11.27	24-Hydroxyl-licorice-saponin A3	C_48_H_72_O_22_	[M−H]^−^	999.4431	999.4385	− 4.61	823.4128, 688.1735	Q/T/X
T2	11.66	Uralsaponin T	C_48_H_74_O_19_	[M−H]^−^	953.4740	953.4710	− 3.19	891.4487, 583.2414	Q/T/X
T3	11.82	Dihydroxy glycyrrhetinic acid	C_42_H_62_O_18_	[M−H]^−^	853.3852	853.3829	− 2.70	504.6233, 351.0551	Q/T/X
T4	11.86	Uralsaponin F	C_44_H_64_O_19_	[M−H]^−^	895.3958	895.3926	− 3.53	797.3072	Q/T/X
T5	11.97	Licoricesaponin A3	C_48_H_72_O_21_	[M−H]^−^	983.4482	983.4441	− 4.21	821.3976, 469.2295	Q/T/X
T6	12.11	Uralsaponin X	C_50_H_74_O_22_	[M−H]^−^	1025.4588	1025.4555	− 3.15	983.4509, 821.3965	Q/T/X
T7	12.63	22β-acetoxyl-Licorice saponin	C_44_H_64_O_18_	[M−H]^−^	879.4008	879.3981	− 3.14	351.8064	Q/T/X
T8/T9	12.72	Licoricesaponin G2/uralsaponin *N*	C_42_H_62_O_17_	[M−H]^−^	837.3903	837.3867	− 4.37	661.3542, 485.8624, 351.0573	Q/T/X
T10	13.42	3-oxo glycyrrhetinic acid	C_30_H_46_O_5_	[M+H]^+^	487.3418	487.3397	− 4.20	469.3297, 451.3188	Q/T/X
T11	13.59	Licoricesaponin E2	C_42_H_60_O_16_	[M−H]^−^	819.3797	819.3765	− 3.94	351.0580, 175.5127	Q/T/X
T12	14.29	18β-Glycyrrhetinic Acid	C_30_H_46_O_4_	[M+H]^+^	471.3468	471.3446	− 4.76	453.3345, 435.3240, 263.1626, 191.1418	Q/T/X
T13	14.31	Glycyrrhizic acid	C42H62O16	[M+H]^+^	823.4110	823.4094	− 1.99	647.3788, 471.3453, 453.3347	Q/T/X
T14	14.61	Licoricesaponin B2	C_42_H_64_O_15_	[M−H]^−^	807.4161	807.4136	− 3.10	351.0919, 175.9585	Q/T/X
T15	14.95	Licoricesaponin R3	C_48_H_74_O_20_	[M−H]^−^	969.4689	969.4653	− 3.69	951.3300, 645.0012	Q/T/X
T16/T17	15.08	Licoricesaponin H2/K2	C_42_H_62_O_16_	[M−H]^−^	821.3954	821.3920	− 4.11	645.3597, 351.0573	Q/T/X
T18	15.62	Uralsaponin C	C_42_H_64_O_16_	[M−H]^−^	823.4110	823.4085	− 3.09	351.0566	Q/T/X
T19	16.09	Uralsaponin W	C_42_H_62_O_15_	[M−H]^−^	805.4004	805.3973	− 3.92	351.0577	Q/T/X
T20	17.63	Glycyrrhetic Acid 3-*O*-Glucuronide	C_36_H_54_O_10_	[M−H]^−^	645.3633	645.3623	− 1.47	469.7497, 175.9586	Q/T/X

### Alkaloids

**Fig. 4:**
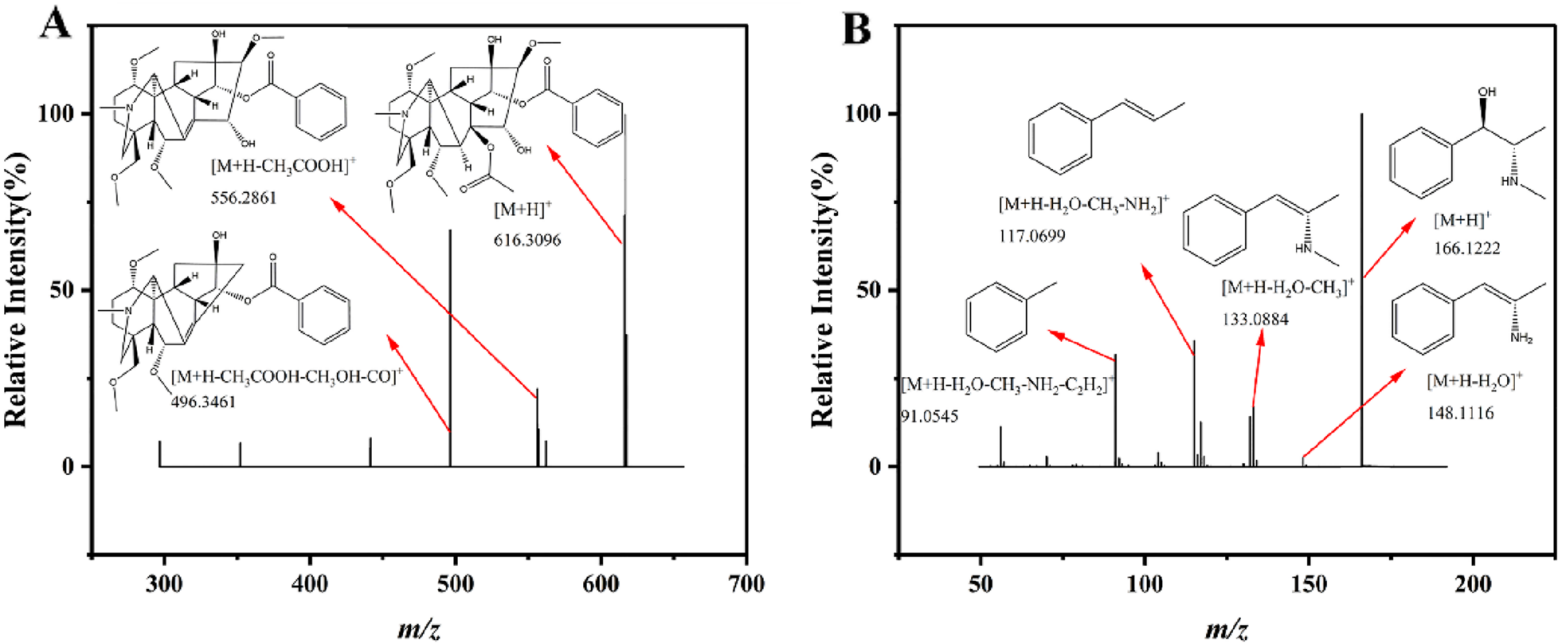
Alkaloids cleavage fragment ions. **A** Hypaconitine. **B** Ephedrine

Alkaloids were the major bioactive compounds identified in the MGF, MGF SA and MIX, presumably from *Ephedra sinica* Stapf and *Aconitum carmichaeli* Debx. For instance, hypaconitine underwent protonation in Fig. 4A, leading to the formation of a precursor ion with *m/z* 616.3096 ([M+H]^+^). Due to the attachment of a proton to the neighboring C atom of the acetoxy group on the C8 side chain, elimination of a CH_3_COOH moiety occurred, producing a *m/z* 556.2861 ([M+H−CH_3_COOH]^+^) ion. Further successive losses of CH_3_OH and CO from the ion of *m/z* 556.2861 resulted in *m/z* 496.3461 ([M+H−CH_3_COOH−CH_3_OH−CO]^+^) ion. The main secondary fragment of the *m/z* 148.1116 ephedrine dehydration peak (Fig. 4B) was fragment ion at *m/z* 133.0884 ([M+H−H_2_O−CH_3_]^+^). The subsequent losses of NH_2_ and C_2_H_2_ generated *m/z* 117.0699 ([M+H−H_2_O−CH_3_−NH_2_]^+^) ion and *m/z* 91.0545 ([M+H−H_2_O−CH_3_−NH_2_−C_2_H_2_]^+^) ion.

### Flavonoids

**Fig. 5:**
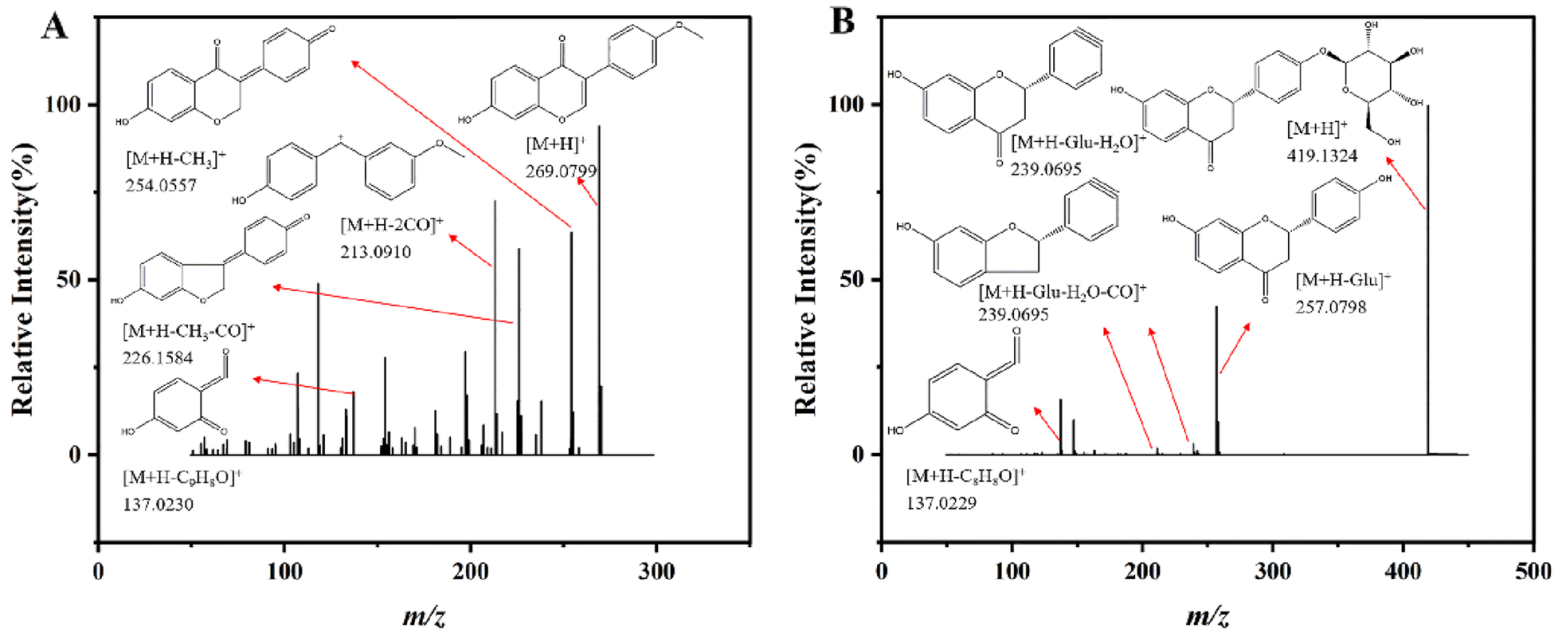
Flavonoids cleavage fragment ions. **A** Formononetin. **B** Liquiritin

Flavonoids with a backbone centered on the 2-phenylchromone skeleton widely found in GC, including formononetin, liquiritin, hesperidin, and others. In Fig. 5A, the precursor [M+H]^+^ ion of formononetin was identified at *m/z* 269.0799. After fragmentation, produced fragment ion at *m/z* 254.0557 ([M+H−CH_3_]^+^) owing to loss CH_3_ on the B ring and rearrange. The subsequent loss of CO led to the formation of [M+H−CH_3_−CO]^+^ fragment ion at *m/z* 226.1584. Additionally, due to the fragmentation of the C ring, a *m/z* 213.0910 ([M+H−2CO]^+^) ion was appeared. And Retro Diels–Alder reaction (RDA) on the C ring produced ion at *m/z* 137.0230 ([M+H−C_9_H_8_O]^+^). Liquiritin played a crucial role as a dihydroflavonoid in MGF. The precursor ion with *m/z* 419.1324 ([M+H]^+^) generated a *m/z* 257.0798 ([M+H−Glu]^+^) ion by eliminating glucose moiety. The subsequent losses of hydroxyl on B ring and carbonyl on C ring gave rise to *m/z* 239.0695 ([M+H−Glu−H_2_O]^+^) ion and 211.0746 ([M+H−Glu−H_2_O−CO]^+^) ion, respectively. The RDA process caused fragmentation production leading to formation of an ion with *m/z* 137.0229 ([M+H−Glu−C_8_H_8_O]^+^) as shown in Fig. 5B.

### Terpenoids

**Fig. 6:**
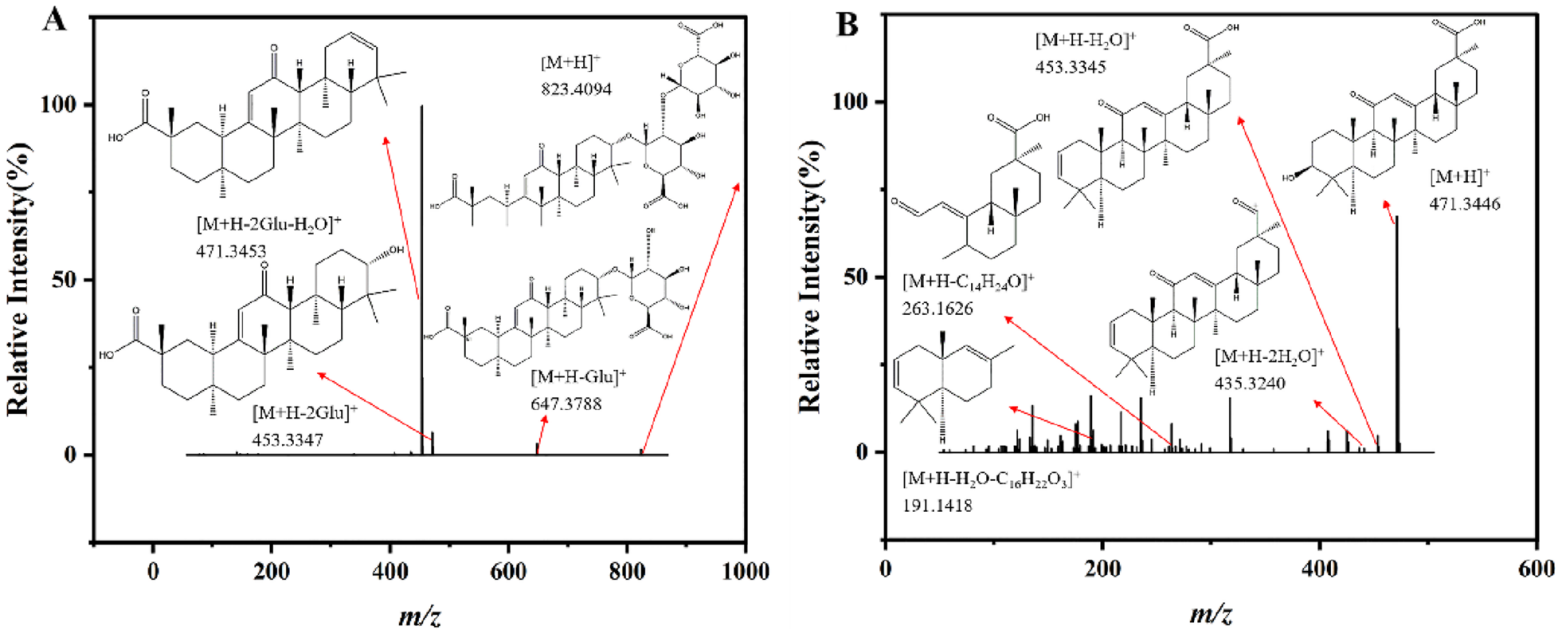
Terpenoids cleavage fragment ions. **A** Glycyrrhizic acid. **B** 18β-Glycyrrhetinic Acid

Triterpene saponins were identified as significant biologically active compounds in MGF, MGF SA and MIX. Glycyrrhizic acid with a protonated form (Fig. 6A) at *m/z* 823.4094 ([M+H]^+^) exhibited a tendency to lost one or two glucuronic acids, resulting in the generation of *m/z* 647.3788 ([M+H−Glu]^+^) and 471.3453 ([M+H−2Glu]^+^) aglycone ions. By shedding H_2_O moieties, these aglycone ions could generate *m/z* 453.3347 ([M+H−2Glu−H_2_O]^+^) ion. The18β-glycyrrhetic acid’s precursor ion peak appeared at *m/z* 471.3446 ([M+H]^+^), which easily lost H_2_O to create peaks at *m/z* 453.3345 ([M+H−H_2_O]^+^) or *m/z* 435.3240 ([M+H−2H_2_O]^+^) as shown in Fig. 6B.

### Effect of MGF, MGF SA and MIX on the activity of RAW264.7 cells and inflammatory activity

**Fig. 7:**
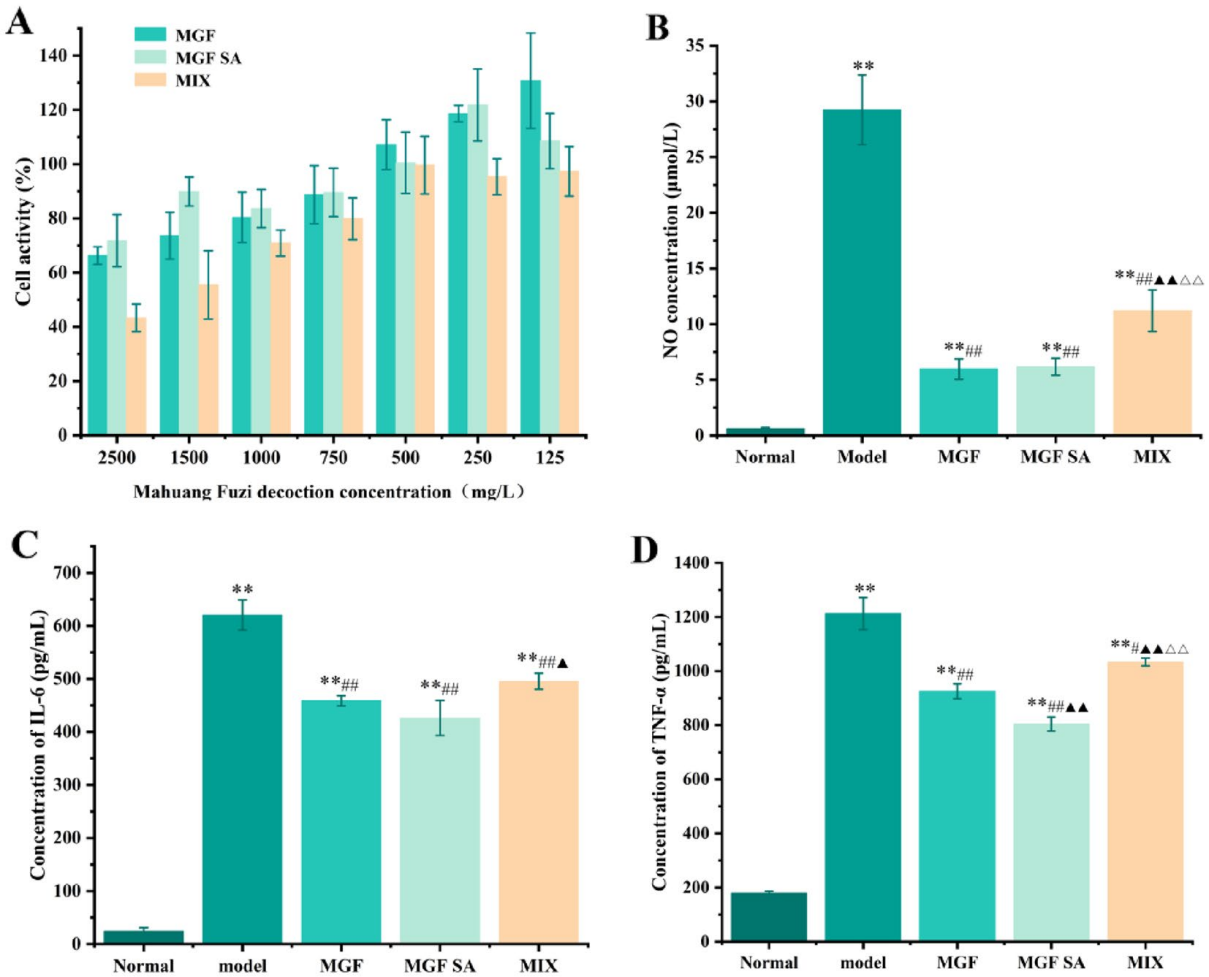
**A** Effects of MGF, MGF SA and MIX on cell activity (*n* = 6). **B** Effect of MGF, MGF SA and MIX on NO concentration (*n* = 6). **C** Effect of MGF, MGF SA and MIX on IL-6 concentration (*n* = 6). **D** Effect of MGF, MGF SA and MIX on TNF-α concentration (*n* = 6). Note: ^**^p < 0.01 compared with normal group; ^#^*p* < 0.05, ^##^*p* < 0.01 compared with model group; ^▲^*p* < 0.05, ^▲▲^*p* < 0.01 compared with MGF group; ^△△^*p* < 0.01 compared with MGF SA group

The cell activity decreased with the increased of the concentration of MGF, MGF SA and MIX (Fig. 7A). When the concentration of MGF, MGF SA and MIX were lower than 500 mg/L, the cell inhibition rate was negative, indicating that samples below this concentration would not inhibit cell growth. Therefore, MGF, MGF SA and MIX with concentrations of 500 mg/L were selected for the following experimental study. LPS, a crucial part of the cell wall of Gram-negative bacteria, could cause macrophages to release a range of inflammatory substances, including NO, IL-6, TNF-α [30, 31]. In this study, ELISA was used to preliminarily study the anti-inflammatory activity of samples with different morphologies and the similar main phytochemicals’ composition. After LPS stimulation, Fig. 7B showed that the contents of NO, TNF-α and IL-6 in control group were significantly lower than those in model group (*p* < 0.01), indicating successful modeling of the model. The contents of NO, IL-6 and TNF-α could be decreased in MGF, MGF SA and MIX. The inhibiting effect of MGF and MGF SA groups on NO and TNF-α concentration were significantly stronger than that of MIX group. The results preliminarily suggested that decoction regulating could improve the activity of samples by influencing the phytochemicals’ morphology.

### Effect of MGF, MGF SA and MIX on NF-κB inflammatory pathway in RAW264.7 cells induced by LPS

**Fig. 8:**
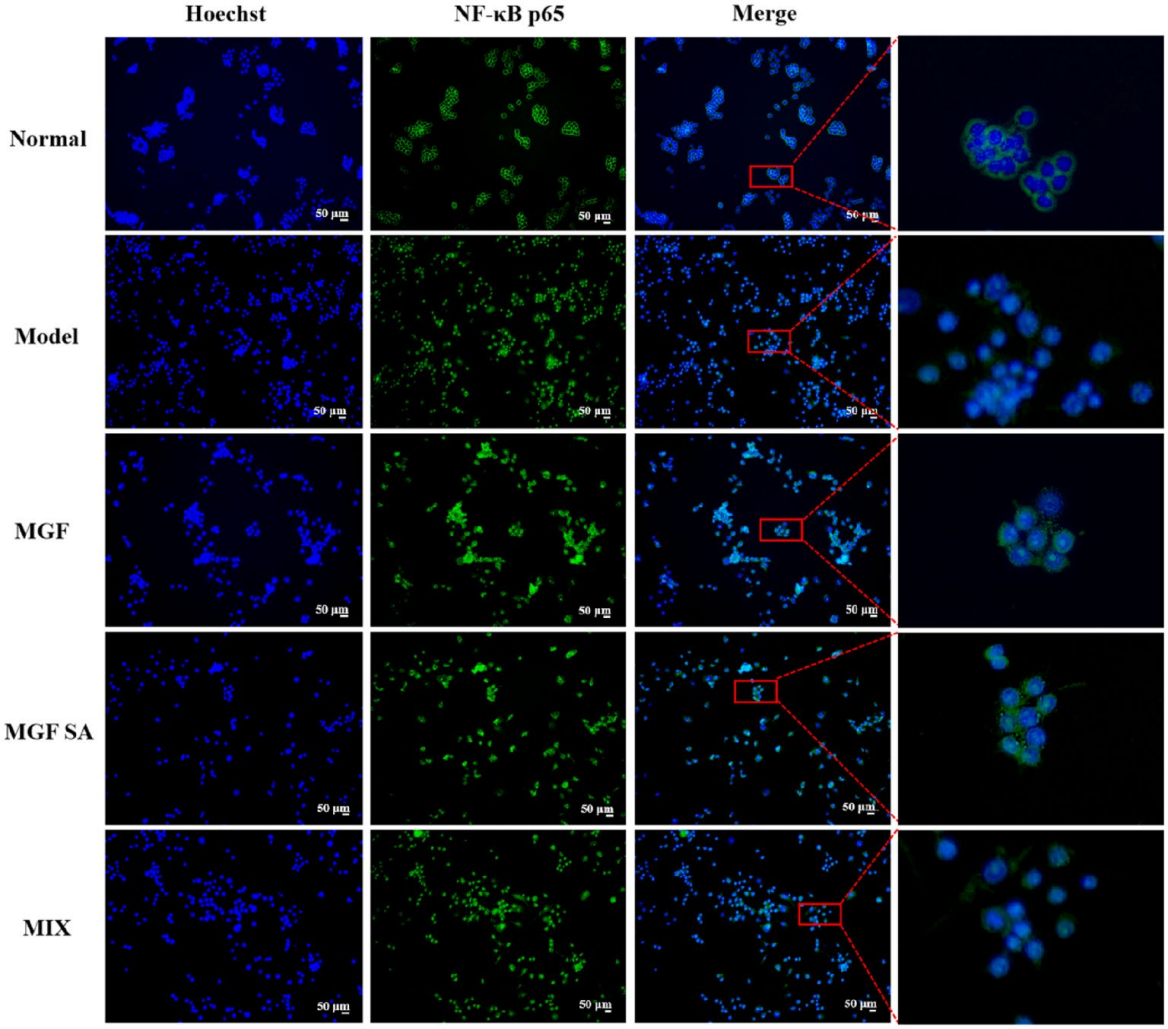
Effects of MGF, MGF SA and MIX on nuclear translocation of NF-κB p65 in LPS-induced RAW 264.7 cells (*n* = 3)

To further evaluate the influence of anti-inflammatory activity of MGF, MGF SA and MIX with different morphology, immunofluorescence was used to observe the expression of NF-κB p65 in nucleus and cytoplasm. It was widely recognized that NF-κB p65, a key transcription regulator, might activate the transcription of cytokines that promote inflammation [32, 33], so the influence of MGF, MGF SA and MIX in activating the NF-κB pathway were investigated. In the normal group, Fig. 8 showed the green fluorescence of NF-κB p65 was mainly observed in the cytoplasm and not in the Hoechst-stained blue nucleus. There was intense green fluorescence in the nucleus, which showed that LPS had caused NF-κB p65 to move from the cytoplasm to the nucleus in the model group. By introducing MGF and MGF SA treatment at a concentration of 500 mg/L, significant inhibition of the LPS-induced NF-κB p65 nuclear migration was possible. Among them, the inhibition effect of MGF SA was more obvious. Then the expression of NF-κB signaling pathway related proteins were determined by western blotting.

**Fig. 9:**
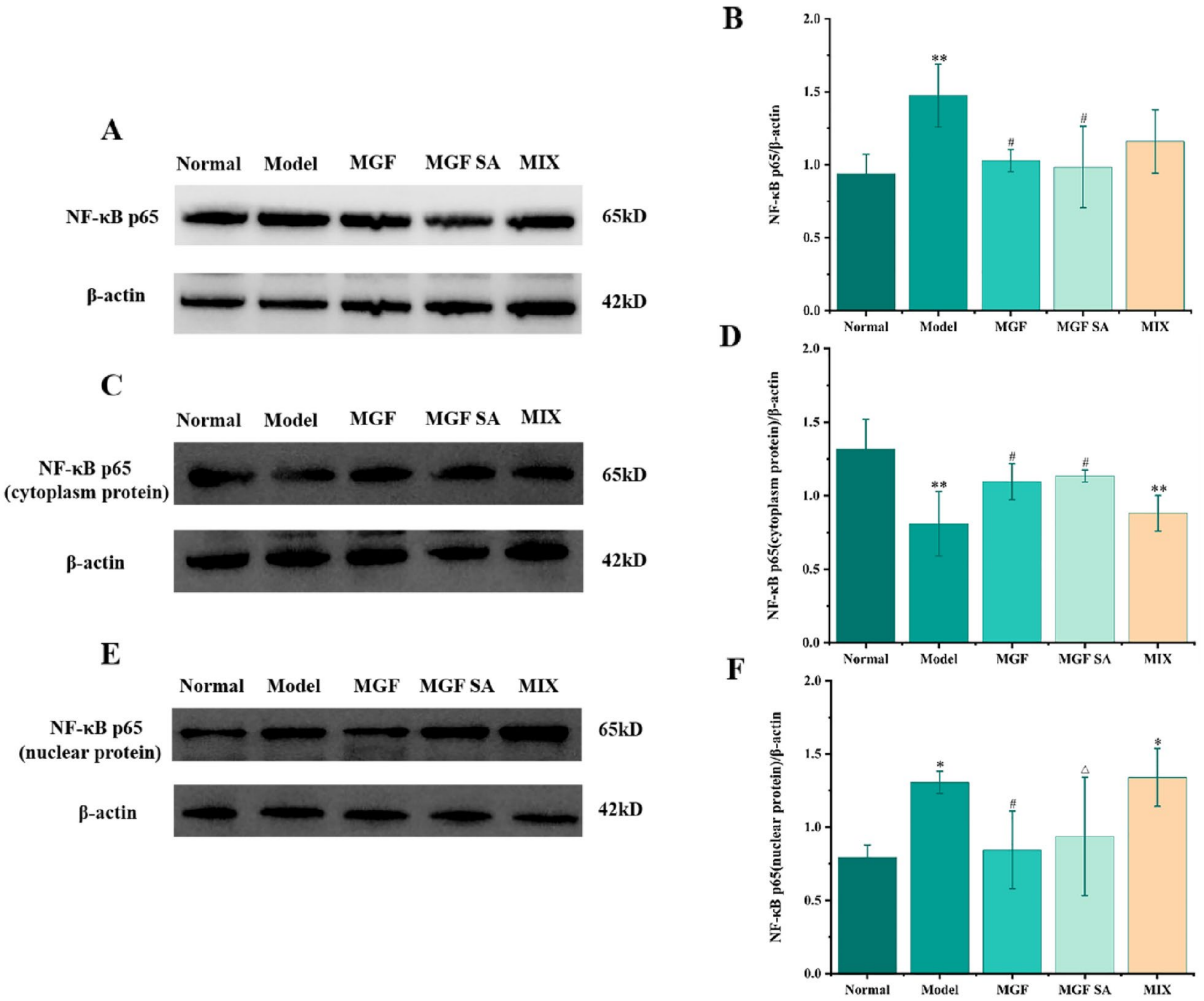
Effects of MGF, MGF SA and MIX on expression of NF-κB p65 total protein, NF-κB p65 nuclear protein and cytoplasmic protein (*n* = 3). Note: ^*^p < 0.05, ^**^p < 0.01 compared with normal group; ^#^*p* < 0.05 compared with model group; ^△^*p* < 0.05 compared with MGF group

LPS stimulation could significantly induce the total protein expression of NF-κB p65 in RAW264.7 cells. MGF and MGF SA at the concentration of 500 mg/L could significantly reduce the total protein expression of NF-κB p65 in inflammatory cells, as shown in Fig. 9A, B. In the normal state of the cells, NF-κB p65 expression in the nucleus was minimal. However, upon LPS stimulation, NF-κB p65 translocated from the cytoplasm to the nucleus, leading to an increase in NF-κB p65 expression in the nucleus and a decrease in its expression in the cytoplasm, as depicted in Fig. 9C–F. Notably, Fig. 9E, F, showed that MGF SA could inhibit the transfer of NF-κB p65 from cytoplasm to nucleus and had a significant difference compared with the MGF group, which was consistent with immunofluorescence results.

### Effect of MGF SA on metabolites in RAW264.7 cells induced by LPS

**Fig. 10:**
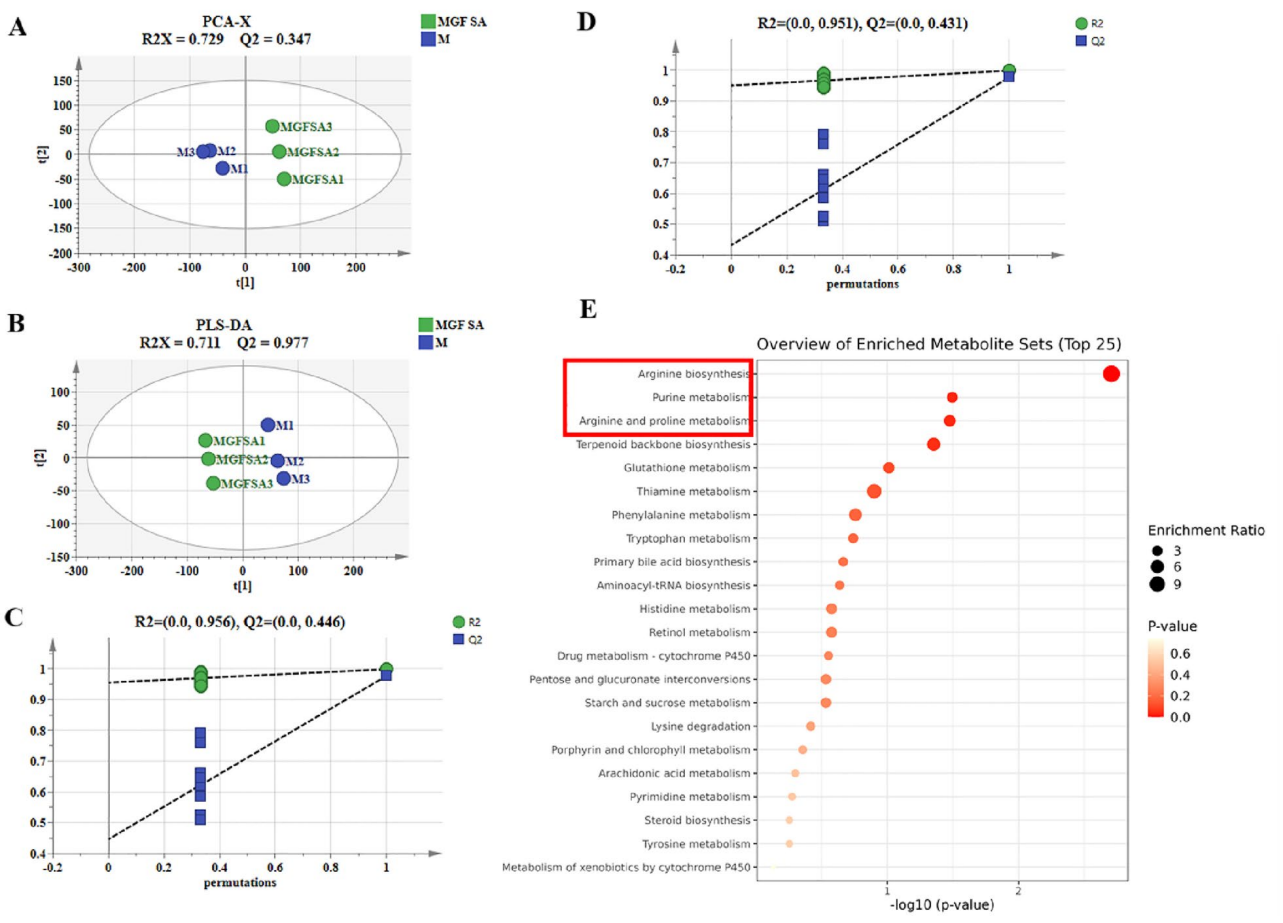
Effects of MGF SA on RAW264.7 inflammatory cells metabolism. **A**, **B** PCA score plot and PLS-DA score plot; **C**, **D** 200-time permutations plots; **E** metabolic pathway enrichment analyses between M and MGF SA)

Metabolomics as a novel field “-omics” technology is a scientific approach for evaluating and researching the mechanisms of herbal medicine [34]. Previous studies had shown that MGF SA could effectively relieve the nuclear metastasis of NF-κB p65 and the release of inflammatory cytokines induced by LPS. Therefore, we further used metabolomics to explore the mechanism of MGF SA intervention on the inflammatory metabolic profile induced by LPS. As shown in Fig. 10A–D, PCA and PLS-DA models were established for statistical analysis of the two groups of metabolic data. It could be seen that PCA and PLS-DA data were separated on both sides of the Y axis, indicating significant differences. R^2^X = 0.729, Q^2^ = 0.347 in PCA model; R^2^Y and Q^2^ of PLS-DA model were 0.999 and 0.977, respectively. The results of permutation test showed that R^2^ and Q^2^ of the MGF SA group and the model group were 0.955 and 0.452. It could be shown that the fit degree and reliability of the two models meet the requirements.

The differential metabolites of the MGF SA group and model (M) group were then examined. A total of 29 differential metabolites were chosen as per the screening criteria (VIP > 1.2, *p* < 0.05), as shown in Table 4. In both healthy and diseased situations, arginine was essential for several metabolic functions, including the urea cycle, synthesis of polyamino acids and creatine, immunological control, and NO synthesis. Studies had shown that inflammation may affect the metabolism of arginine, and abnormal metabolism of arginine and proline may exacerbate inflammation [34–36]. Inflammatory responses stimulate metabolism and cells degrade AMP into hypoxanthine nucleotides, which in turn degrade into inosine, hypoxanthine, and urate, which could lead to arthritis [37]. The probable metabolic pathways were further examined based on the different metabolites. The results of metabolic pathway analysis in this study were of great significance for the study of the mechanism of MGF SA. As shown in Fig. 10D, the metabolism of arginine biosynthesis (*p* < 0.01), purine metabolism (*p* = 0.02), arginine and proline metabolism (*p* = 0.03) and terpenoid backbone biosynthesis (*p* = 0.04) were significantly affected in RAW264.7 inflammatory cells. Therefore, it was preliminary suggested that MGF SA acted as an anti-inflammatory by regulating the metabolic pathways of arginine, proline and purine.

**Table 4:** The MGF SA affect the differential metabolites of RAW264.7 inflammatory cells metabolism

HMDB ID	Metabolite	Formula	t_R_/min	Ionic Form	Mass Discrepancy	*p*	VIP	Trend	KEGG
HMDB0012452	All-trans-18-Hydroxyretinoic acid	C_20_H_28_O_3_	15.86	[M+H]^+^	0.0007	0.0000	1.41	Up	Retinol metabolism
HMDB0001343	Mevalonic acid-5P	C_6_H_13_O_7_P	7.73	[M+H_2_O+H]^+^	0.0002	0.0014	1.45	Up	Terpenoid backbone biosynthesis
HMDB0000291	Vanillylmandelic acid	C_9_H_10_O_5_	1.55	[M+C_3_H_4_O_2_+H]^+^	0.0001	0.0012	1.40	Down	Tyrosine metabolism
HMDB0060651	2-Hydroxycarbamazepine	C_15_H_12_N_2_O_2_	5.37	[M+CO_2_+H]^+^	0.0000	0.0001	1.42	Up	Drug metabolism—cytochrome P450
HMDB0000870	Histamine	C_5_H_9_N_3_	0.77	[M+H]^+^	0.0004	0.0000	1.42	Up	Histidine metabolism
HMDB0029751	Naphthalene	C_10_H_8_	7.56	[M]^+^	0.0002	0.0000	1.59	Down	Metabolism of xenobiotics by cytochrome P450
HMDB0000014	Deoxycytidine	C_9_H_13_N_3_O_4_	2.36	[M(C13)+2H]^2+^	0.0003	0.0000	1.22	Down	Pyrimidine metabolism
HMDB0000214	Ornithine	C_5_H_12_N_2_O_2_	0.92	[M+NH_3_+H]^+^	0.0002	0.0000	1.26	Down	Arginine biosynthesis; arginine and proline metabolism; glutathione metabolism
HMDB0000159	l-Phenylalanine	C_9_H_11_NO_2_	1.50	[M−HCOOH+H]^+^	0.0001	0.0037	1.31	Down	Phenylalanine metabolism; aminoacyl-tRNA biosynthesis
HMDB0000904	Citrulline	C_6_H_13_N_3_O_3_	0.91	[M−HCOOH+H]^+^	0.0000	0.0000	1.23	Down	Arginine biosynthesis
HMDB0000034	Adenine	C_5_H_5_N_5_	0.95	[M+H]^+^	0.0001	0.0003	1.39	Down	Purine metabolism
HMDB0000510	Aminoadipic acid	C_6_H_11_NO_4_	1.47	[M−H_2_O+H]^+^	0.0002	0.0122	1.37	Down	Lysine degradation
HMDB0242149	Xylitol	C_5_H_12_O_5_	0.91	[M]^+^	0.0004	0.0395	1.38	Down	Pentose and glucuronate interconversions
HMDB0000517	l-Arginine	C_6_H_14_N_4_O_2_	0.89	[M−NH_3_+H]^+^	0.0000	0.0000	1.23	Down	Arginine biosynthesis; Arginine and proline metabolism; Aminoacyl-tRNA biosynthesis
HMDB0001123	2-Aminobenzoic acid	C_7_H_7_NO_2_	0.87	[M+Na]^+^	0.0006	0.0016	1.25	Down	Tryptophan metabolism
HMDB0060389	4-Hydroxy-5-phenyltetrahydro-1,3-oxazin-2-one	C_10_H_11_NO_3_	6.29	[M-NH_3_+H]^+^	0.0001	0.0002	1.21	down	Drug metabolism—cytochrome P450
HMDB0000175	Inosinic acid	C_10_H_13_N_4_O_8_P	3.67	[M+H+Na]^2+^	0.0000	0.0011	1.30	Up	Purine metabolism
HMDB0004224	*N*(omega)-hydroxyarginine	C_6_H_14_N_4_O_3_	0.80	[M+H]^+^	0.0004	0.0452	1.21	Up	Arginine and proline metabolism
HMDB0000763	5-Hydroxyindoleacetic acid	C_10_H_9_NO_3_	5.08	[M+H]^+^	0.0001	0.0000	1.23	Down	Tryptophan metabolism
HMDB0000245	Porphobilinogen	C_10_H_14_N_2_O_4_	0.93	[M+H_2_O+H]^+^	0.0002	0.0002	1.30	Down	Porphyrin and chlorophyll metabolism
HMDB0000050	Adenosine	C_10_H_13_N_5_O_4_	1.17	[M+H]^+^	0.0000	0.0000	1.34	Up	Purine metabolism
HMDB0001049	gamma-Glutamylcysteine	C_8_H_14_N_2_O_5_S	7.55	[M+H_2_O+H]^+^	0.0006	0.0001	1.23	Down	Glutathione metabolism
HMDB0000235	Thiamine	C_12_H_17_N_4_OS	1.27	[M+Na]^+^	0.0001	0.0004	1.23	Up	Thiamine metabolism
HMDB0001517	AICAR	C_9_H_15_N_4_O_8_P	0.92	[M−HCOOH+H]^+^	0.0014	0.0001	1.26	Down	Purine metabolism
HMDB0001198	Leukotriene C4	C_30_H_47_N_3_O_9_S	2.01	[M(C13)+2H]^2+^	0.0008	0.0000	1.22	Down	Arachidonic acid metabolism
HMDB0000163	d-Maltose	C_12_H_22_O_11_	0.92	[M+Na]^+^	0.0000	0.0000	1.24	Down	Starch and sucrose metabolism; Starch and sucrose metabolism
HMDB0000961	Farnesyl pyrophosphate	C_15_H_28_O_7_P_2_	9.82	[M+H_2_O+H]^+^	0.0001	0.0000	1.20	Down	Terpenoid backbone biosynthesis; Steroid biosynthesis
HMDB0001993	7a-Hydroxy-cholestene-3-one	C_27_H_44_O_2_	18.72	[M+H]^+^	0.0002	0.0026	1.37	Down	Primary bile acid biosynthesis
HMDB0002103	27-Hydroxycholesterol	C_27_H_46_O_2_	15.19	[M+HCOONa]^+^	0.0019	0.0000	1.20	Down	Primary bile acid biosynthesis

## Conclusions

In this study, it was found that the morphology of MGF co-decoction was more uniform than that of the physical mixture of single decoction. Meanwhile, the MGF SA extracted from co-decoction displayed spherical nanoparticles. Interestingly, though the micromorphology was different, the comparison of phytochemicals of the co-decoction and physical mixing were roughly the same. Anti-inflammatory evaluation showed that MGF and MGF SA had better activity than MIX; and MGF SA had a significant effect on improving nuclear transfer of NF-κB p65 than other groups, suggesting that supermolecules were the key component of the pharmacodynamic contribution of MGF decoction. LPS caused a series of metabolic pathways of RAW264.7 cells to be disordered, and the disordered metabolic pathways were effectively regulated after MGF SA intervention. The mechanism might be connected to MGF SA’s control of arginine biosynthesis, purine metabolism, arginine and proline metabolism. In summary, MGF, MGF SA and MIX had the similar main phytochemicals’ composition and administered at the same dose, but diverse biological effects were produced by their varied molecular morphologies. This work exhibited that decoction could regulate phytochemicals’ micromorphology and anti-inflammation activity of supermolecules originated from MGF decoction. In addition, current study displayed that the supermolecules as one of the main pharmacodynamic site of herb medicine decoction would be a new hotspot in future research and co-decocting herb might be more beneficial to the treatment of diseases than the mixture of the single herbs’ extraction.

## Data Availability

The datasets generated or analysed during this study are available from the corresponding author on reasonable request.

## Abbreviations

DLS: Dynamic light scattering
FESEM: Field emission scanning electron microscopy
FZ: *Aconitum carmichaeli* Debx
GC: *Glycyrrhiza uralensis* Fisch
ITC: Isothermal titration calorimetry
MGF: Mahung Fuzi decoction
MGF SA: MGF supermolecules
MH: *Ephedra sinica* Stapf
MIX: Physical mixture of MGF single decoction
NF-κB: Nuclear transcription factor-κB
NPs: Nanoparticles
UHPLC-Q-Orbitrap HRMS: Ultra high performance liquid chromatography-Q Exactive hybrid quadrupole-orbitrap high-resolution accurate mass spectrometry
